# On Regulating Teeth

**Published:** 1883-06

**Authors:** Nathaniel Tracy

**Affiliations:** Ipswich


### On Regulating Teeth.

BY NATHANIEL TRACY, L.D S. ENG., Ipswich.*

MR. PRESIDENT AND GENTLEMEN:—It is not my intention to enter into a lengthened exposition of the subject of the regulation of teeth, but rather to state as plainly and concisely as I can the conclusions I have attained as to the best methods of practice in this department of our profession.

The importance of the subject cannot be denied. The advantages of a good, well-arranged set of teeth, as conducing to their usefulness and the greater probability of their preservation, as well as their effect on the appearance, must be acknowledged by all. The latter consideration, the effect upon the appearance, especially as it affects the gentler sex, should not be lost sight of, as the expression, natural and becoming to a face, may be marred if the teeth are either allowed or caused to protrude, or to be unfairly contracted. So that it is very necessary, in the consideration of the course to be pursued in the regulation of the teeth, to consider their position, not in relation to the oral cavity alone, but also their effect on the fair proportions of the face.

With regard to the usual irregularities, I shall broadly and simply divide them into two great divisions—malposition of the teeth in fairly developed maxillæ, and malposition and defective antagonism of teeth consequent on irregularities in the development of the maxillæ.

The treatment of the first class must depend on the period at which the case comes under our notice; early attention to direct-

* Read before the Eastern Counties Dental Association, at the Annual General Meeting, held at Ipswich, on the 11th inst.

ing the incisors into proper position will often prevent much future trouble. And here I would oppose the practice of removing the contiguous teeth, but would rather at once attempt to expand the arch to give room for the permanent teeth, unless the considerations previously mentioned indicated advantages to be gained by the contraction. Should it be necessary to use mechanical means, I prefer to make the plate of rubber, vulcanized on a tin model to ensure sharpness of fit, with metal biting surfaces struck up to fit opposing bite when they are required to be worn for any length of time, or entirely of metal when the case is more complicated. When the correction required is to advance any of the teeth, I give the preference, if only one or a few are affected, to the use of a spring formed of gold wire with a free end. But when the alteration extends to many, I have found a bar fitted to the plate and extended in front, to which elastic rings can be secured, a very convenient form, and rapid in action. When isolated teeth, require to be retracted, I use elastic rings attached to the plate, with, if necessary, springs to give direction; but where the front teeth generally require that treatment, I have found a loose bar fitted to the teeth, and secured to the plate by elastic rings, brings them into position very easily, afterwards retaining them by a fixed bar. A common defect we meet with, is where the cuspids are made to protrude by their proper space being filled up by the bicuspids. Usually, if the first permanent molars are affected by caries, as in most cases they are, their removal will remedy the mischief. But in these cases, I usually defer their removal until the second molars are fairly antagonized. Should the bicuspids prove obstinate, and resist the pressure of the cuspids, they can then be retracted by elastic bands fastened to a plate carried by the second molars. But should the teeth be all healthy, I should remove the first bicuspids to give immediate relief, but should then prefer to treat the opposing teeth symmetrically, unless there should be a special indication for a different policy.

The other class of cases of defective development of the maxillæ, shown principally in the contracted upper maxilla, I have corrected by the expanding plate. This I usually make with a gold spring. The very fact of increasing the width, often allows the pressure of the lip to flatten the arch in front. If not, it is easy, after having obtained the space by the expansion, to draw them into position by the loose bar, as before mentioned, afterwards retaining them by a fixed bar.

Another defect is sometimes caused by the want of development in the ramus of the lower jaw causing the inferior incisors to strike closely against the superior and force them forward. This I have corrected by a plate fitted to the temporary molars, so as to prevent antagonism in the molars of the permanent set; at the same time leaving the superior incisors free from any pressure of the plate. As the permanent molars elongate, if the superior incisors are not retracted by the muscular action of the lip, I use the band as in other cases.—*The Journal of the British Dental Association.*

